# Ameliorating Effect of *Glehnia littoralis* Extract on Periodontitis Through Regulation of 11β-Hydroxysteroid Dehydrogenase Type 1 in an Experimental Periodontitis Model

**DOI:** 10.3390/molecules30142903

**Published:** 2025-07-09

**Authors:** Eun-Nam Kim, Nguyen Minh Trang, Chae Lee Park, Sang-Yoon Kim, MinKyun Na, Gil-Saeng Jeong

**Affiliations:** 1 College of Pharmacy, Chungnam National University, Daejeon 34134, Republic of Korea; enkim@cnu.ac.kr (E.-N.K.); ngminhtrang52@gmail.com (N.M.T.); mkna@cnu.ac.kr (M.N.); 2 Healthcare Research Division, HuonsGlobal Bldg., Pangyo 13449, Republic of Korea; cherry890709@huons.com (C.L.P.); sykim78@huons.com (S.-Y.K.)

**Keywords:** anti-inflammation, *Glehnia littoralis*, osteoblast, human periodontal ligaments cells, 11β-HSD1, periodontitis

## Abstract

*Glehnia littoralis* Fr. Schmidt ex Miq. has been cultivated in China for a long time and used as a medicinal plant called “Beishashen” in traditional Chinese medicine and has been traditionally known to have antibacterial and anti-inflammatory effects, but its direct role in periodontitis has not been known. Currently used periodontal treatments require long-term administration, which causes many side effects. Therefore, in this study, we evaluated the effects of *G. littoralis* extract (GLE) on periodontitis in an experimental periodontitis-induced in vitro and *vivo* model and understood its potential molecular mechanism. The effect of GLE on periodontitis in vitro was investigated using human periodontal ligament (HPDL) cells mediated by PG-LPS. Additionally, a ligature-induced periodontitis model and a PG-LPS-induced periodontal inflammation model were used to investigate the effect of GLE in vivo. In vitro study results showed that GLE down-regulated the increased inflammatory cytokines and mediators in HPDL cells stimulated with PG-LPS, and simultaneously down-regulated the levels of 11β-HSD1 and glucocorticoid receptor (GR), thereby alleviating periodontal inflammation. At the same time, it restored the lost osteoblast differentiation potential of HPDL cells. In addition, in an in vivo model representatively used for periodontitis research, the periodontal inflammation-alleviating effect and the effect of restoring or protecting damaged periodontal tissue were confirmed. GLE can be considered as a new periodontitis treatment agent through regulating 11β-HSD1.

## 1. Introduction

According to the National Dental and Craniofacial Institute of the United States, it has been found that periodontal disease occurs through tartar, a sticky film of bacteria commonly known as plaque, which accumulates and forms on teeth; periodontal disease has many forms and symptoms, with gingivitis and periodontitis being the most common [1]. Among them, periodontitis is known as the second most common type of periodontal disease worldwide after tooth decay, and individual oral hygiene problems, smoking, and dietary problems are known to be the main causes of periodontitis, though various other complex problems are also causes [2]. Periodontitis begins with initial inflammation, and this long-term chronic inflammation affects the bone and bearing capacity of periodontal tissue; it is characterized by pocket spaces created between loose teeth and gums [3]. Most of the treatments for periodontitis are to physically remove bacterial manipulations and calculus through the use of surgical manual devices or ultrasonic devices, which are successful for most patients, but may not be appropriate for some patients. It can cause severe pain and poor prognosis [4]. Another treatment option for periodontitis is pharmacological therapy based on host control, such as oral microbes or bacteria, using probiotics and antibacterial agents [5,6]. However, although these pharmacological therapies have recently attracted a lot of attention, they have the disadvantage of being ineffective for patients and requiring long-term use. Therefore, studies are being actively conducted to obtain alternatives from safe natural products that are relatively free from these problems [7,8].

Among the enzymes involved in the biosynthesis and decomposition of glucocorticoids, 11β-hydroxysteroid dehydrogenase type 1 (11β-HSD1) is known to be involved in metabolic diseases including obesity and diabetes and is known to play a role in catalyzing the conversion to active glucocorticoids [9,10]. Recent studies have reported clinical results showing that 11β-HSD1 is increased in patients with periodontitis, and in fact, serum and salivary cortisol levels are known to be correlated with chronic periodontitis [11]. In particular, some patients with periodontitis have metabolic diseases such as diabetes, and it has been reported that diabetic patients have specific polymorphisms or mutations in genes encoding interleukin, which may make inflammatory diseases more severe [12]. In addition, 11β-HSD1 was known to contribute to the induction of inflammatory gene expression or activation of immunosuppressive genes through binding to the glucocorticoid receptor (GR) and the activation and increase in target receptors such as Angptl4 [13]; but recent research results have shown that the conversion of cortisol by the increase of 1β-HSD1 is known to amplify inflammatory and oxidative stress responses by activating the NF-κB and MAPK signaling pathways, and conversely, mitogen-activated protein kinase (MAPK) pathways such as p38 and ERK are also known to affect inflammation and cell damage through 11β-HSD1 gene activation [14]. Given these points, 11β-HSD1 is currently considered a useful therapeutic target not only in metabolic diseases but also in chronic inflammatory diseases, and in research reports on periodontitis; the expression of 11β-HSD1 was detected in oral fibroblasts and keratinocytes, but its role in periodontitis and periodontal ligament is still unclear [15,16].

Periodontal ligament (PDL), which is composed of fibroblasts, osteoblasts, and cementoblasts is located between the cementum and the alveolar bone and supports the periodontal connective tissue; it is known to be a very important fibroblast, playing the role of periodontal ligament cells that form the periodontal ligament [17,18,19]. Several previous studies have shown that promoting cell proliferation and osteogenic differentiation of human periodontal ligament cells (HPDLC) to regenerate periodontal tissue has a very positive effect on the treatment of periodontal disease; it has been reported to play a very important role in regulating the length of the periodontal crest or the amount of lost alveolar bone [20]. Therefore, the recovery of periodontal ligament cells damaged by periodontitis is very important for periodontal health.

*Glehnia littoralis* Fr. Schmidt ex Miq. is a deep-rooted perennial plant that grows mainly in temperate coastal sand dunes and is classified as a halophyte; it is a medicinal plant that has been cultivated in China for a long time, although it grows in areas with high salt concentrations in soil where general land plants cannot grow [21,22]. *G. littoralis* is called “Beishashen” in traditional Chinese medicine and has traditionally been known to have antibacterial and anti-inflammatory effects, but its direct role in periodontitis has not been known [23]. The main components of *G. littoralis* contain various coumarins and alkaloids, and in addition, they have been studied to have various physiological activities such as antifungal, anti-obesity, and antioxidant effects [24,25,26]. In addition, the lotus leaf of *G. littoralis* is mainly used as raw vegetables or herbs such as leafy vegetables and mountain vegetables, and the leaves and stems are used for manufacturing processed products such as makgeolli, rice cakes, tea, and extracts, and it is a familiar food material listed in the Food Codex of South Korea. However, despite the physiological activity and commercial efficiency of *G. littoralis*, research is still incomplete. Therefore, in this study, we propose the possibility of *G. littoralis* as a periodontitis regulator by evaluating the effect of *G. littoralis* extract (GLE) on periodontitis in human periodontal ligament cells stimulated with *Porphyromonas gingivalis*-lipopolysaccharides (PG-LPS) and periodontitis-induced in vivo experimental animal model.

## 2. Results

### 2.1. Major Component Analysis of GLE

**Figure 1:**
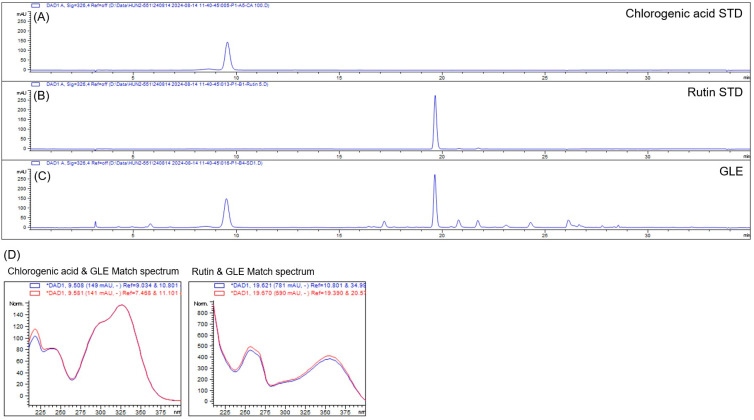
Analysis of major components in GLE. HPLC chromatogram of (**A**) chlorogenic acid standard, (**B**) rutin standard, and (**C**) GLE. (**D**) Each peak match spectrum. The detection wavelength was 326 nm.

Prior to this study, the main components of GLE were analyzed by HPLC-DAD to identify them. As a result of the analysis, chlorogenic acid (Figure 1A) and rutin (Figure 1B) were identified as the main components of GLE (Figure 1C), and it was confirmed that the peak wavelengths contained in GLE and the peak wavelengths of each component were identical (Figure 1D). In addition, it was confirmed that these results were consistent with the results of the previously reported GLE component analysis study [26]. In addition, the two components detected from GLE were quantitatively analyzed by HPLC-DAD, and the linearity of the standard was 0.99 or higher, and chlorogenic acid was 7.040 ± 0.041 mg/g and rutin was 20.194 ± 0.004 mg/g (Table 1).

### 2.2. The Evaluation of Cytotoxicity on GLE in HPDL Cells

**Figure 2:**
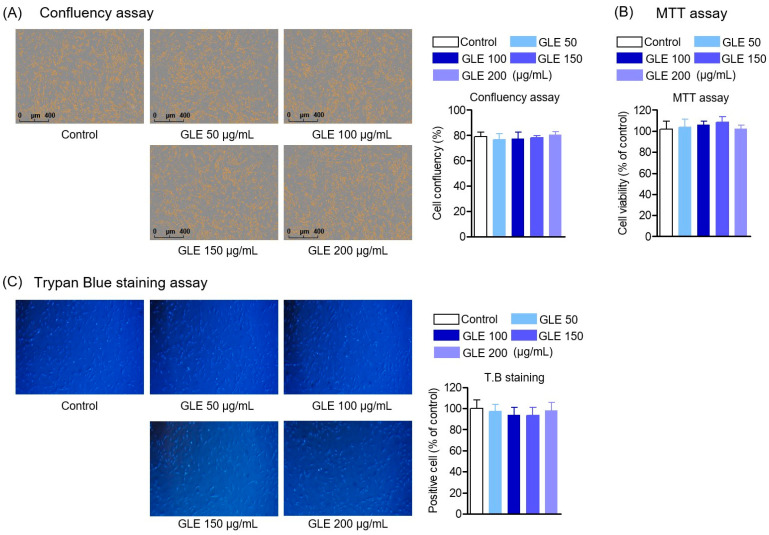
The evaluation of cytotoxicity on GLE in HPDL cells. HPDL cells were seeded in 96-well plates at a density of 5 × 10^2^ cells/well, cultured for 24 h, and then treated with the indicated concentrations of GLE for 7 days. (**A**) Cells were then counted using the Incucyte^®^ Live-Cell Analysis System (Göttingen, Germany). When counting cells, the cell numbers of the stained control group were converted to percentages and averaged for the GLE-treated group. (**B**) In addition, an MTT assay was performed using the same GLE treatment method, and (**C**) trypan blue staining was performed to count the number of positive cells, convert the number of cells in the control group into a percentage, and average the number of cells in the GLE treatment group.

To evaluate the cytotoxicity of GLE on HPDL cells, MTT assay, cell confluency assay, and trypan blue staining were performed. GLE at the indicated concentrations (50, 100, 150, and 200 μg/mL, respectively) were treated for 7 days. The results showed that GLE did not affect the morphological transformation and counting of cells (Figure 2A) and did not show cytotoxicity in the MTT assay (Figure 2B). In addition, trypan blue staining results confirmed that it did not affect cell toxicity. Therefore, subsequent experiments were performed within the concentration range of GLE 50 to 200 μg/mL.

### 2.3. GLE Regulates 11β-HSD1 Produced by PG-LPS

**Figure 3:**
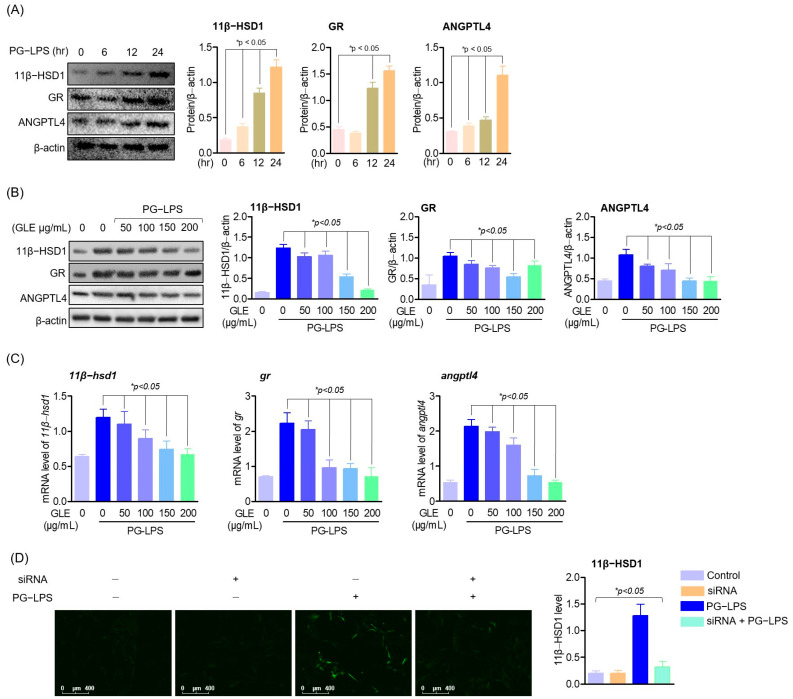
GLE regulates 11β-HSD1 produced by PG-LPS. HPDL cells were seeded in 6-well plates at 5 × 10^5^ cells/well and treated with GLE at the indicated concentrations for the indicated times. (**A**) Western blot was performed after 6, 12, and 24 h of PG-LPS treatment. (**B**) Western blot was performed after 6 h of GLE treatment at the indicated concentrations and 24 h of PG-LPS stimulation. (**C**) After treating the cells using the same experimental method, the expression of each mRNA was measured by real-time PCR. (**D**) After silencing 11β-HSD1 with siRNA, PG-LPS was treated for 24 h, and FITC staining was performed. Data are expressed as mean ± SD. * *p* < 0.05, compared with the group treated with PG-LPS only.

First, in this study, to evaluate the effect of GLE on regulating 11β-HSD1, the effect of GLE on the production of 11β-HSD1 produced by PG-LPS treatment, GR, known as a glucocorticoid receptor, and Angptl4, known as the target receptor of glucocorticoid, was examined. Research results showed that the expression of 11β-HSD1 and the protein expression of receptor GR and Angptl4 were induced depending on the treatment time of PG-LPS in HPDL cells (Figure 3A); it was confirmed that the level of expression of these proteins was down-regulated by treatment with GLE (Figure 3B). In addition, to explore the effect of GLE on these gene levels, the gene levels were analyzed through qPCR. As a result, GLE treatment similarly down-regulated the levels of *11β-hsd1*, *gr*, and *angptl4* in a concentration-dependent manner (Figure 3C). Therefore, we aimed to evaluate the effect of GLE on regulating 11β-hsd1 in periodontitis by silencing 11β-hsd1 using siRNA. First, we attempted to secure a research model by silencing 11β-hsd1 in HPDL cells. The data confirmed that 11β-hsd1 was produced by PG-LPS, but its expression was knocked down when silenced with siRNA (Figure 3D). From these results, it was confirmed that GLE downregulates 11β-HSD1 produced by Pg-LPS and further suggests its potential as a periodontitis regulator through its role as an 11β-HSD1 regulator.

### 2.4. GLE Regulates MAPK Phosphorylation

**Figure 4:**
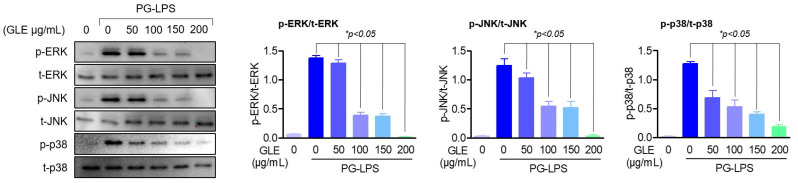
GLE regulates MAPK phosphorylation. GLE regulates M0APK phosphorylation. HPDL cells were seeded in 6-well plates at 5 × 10^5^ cells/well and treated with the indicated concentrations of GLE for 4 h, followed by PG-LPS treatment for 1 h. Western blot was performed after 1 h of PG-LPS treatment. Data are expressed as mean ± SD. * *p* < 0.05, compared with the group treated with PG-LPS only.

PG-LPS is known to induce inflammatory responses in various cell types, and, in particular, to increase the expression of inflammatory cytokines through the phosphorylation of MAPK signaling pathways [12]. Furthermore, according to reports known to date, the activation of MAPK signaling pathways is known to have a mutually complementary effect on the process of cortisol conversion by 11β-HSD1 increase [13]. Therefore, we confirmed the effects of GLE on the MAPK signaling pathways. These results showed that GLE dose-dependently down-regulated the expression of phosphorylated proteins of ERK, JNK, and p38 among MAPKs. These results suggest that GLE may be involved in the MAPK signaling pathway in the 11β-HSD1 production process and may exhibit anti-inflammatory effects through inhibition of the cortisol conversion process (Figure 4).

### 2.5. The Anti-Inflammatory Effect of GLE Is Achieved Through the Regulation of 11β-HSD1

**Figure 5:**
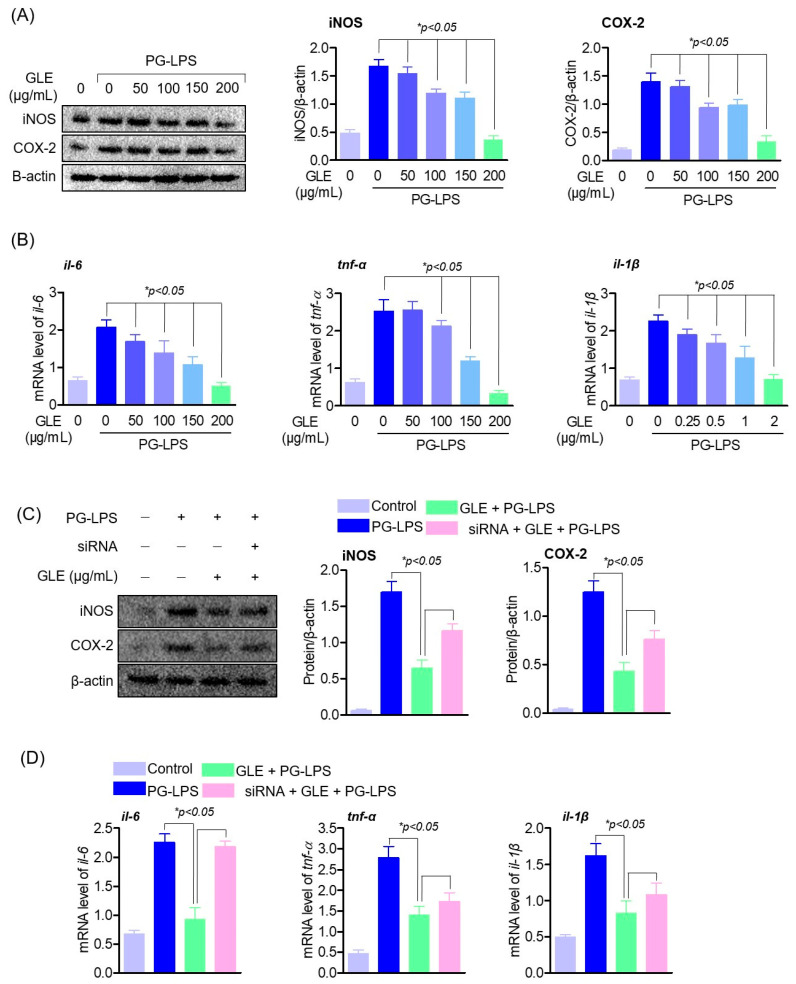
The anti-inflammatory effect of GLE is achieved through regulation of 11β-HSD1. (**A**) HPDL cells were seeded at 5 × 10^5^ cells/well in 6-well plates and treated with the indicated concentrations of GLE (50, 100, 150, and 200 µg/mL) for 6 h and PG-LPS for 24 h, and then Western blot was performed. (**B**) The mRNA levels of inflammatory cytokines (il-6, il-1β, and tnf-α) were analyzed by real-time PCR after treating the samples and PG-LPS in the same manner. (**C**) Western blot was performed after treatment with 200 µg/mL GLE with or without silencing 11β-HSD1 with siRNA. (**D**) mRNA levels of inflammatory cytokines (*il-6*, *il-1β*, and *tnf-α*) were analyzed by real-time PCR analysis after treatment with 200 µg/mL GLE with or without silencing 11β-HSD1 in the same manner. Data are expressed as mean ± SD. * *p* < 0.05, compared with the group treated with PG-LPS only or GLE + PG-LPS treated group.

First, to evaluate the inhibitory effect of GLE on periodontitis, the modulating effect of GLE on pro-inflammatory mediators in HPDL cells stimulated with PG-LPS was evaluated using Western blot assay. As a result of the study, GLE down-regulated the expression levels of pro-inflammatory mediator proteins increased by PG-LPS in a concentration-dependent manner (Figure 5A), and increased gene levels of *il-6*, *il-1β*, and *tnf-α*, the major pro-inflammatory cytokines in periodontitis (Figure 5B). Therefore, to evaluate the effect of 11β-HSD1 on the anti-inflammatory effect of GLE, 11β-HSD1 was silenced in HPDL cells through siRNA treatment. As a result, it was confirmed that the previously shown down-regulation effect of GLE’s pro-inflammatory mediator protein and major pro-inflammatory cytokines of periodontitis was reversed (Figure 5C,D). These results suggest the possibility of GLE as a regulator of periodontitis through its anti-inflammatory effect in HPDL cells, and it appears that this effect may result from the regulation of 11β-HSD1. Therefore, in the subsequent study, an experiment was conducted to determine what role 11β-HSD1 silencing would play in the efficacy of GLE in periodontal inflammation.

### 2.6. GLE Regulates the Osteoblastic Differentiation Capacity of HPDL Cells Lost by PG-LPS Through 11β-HSD1

**Figure 6:**
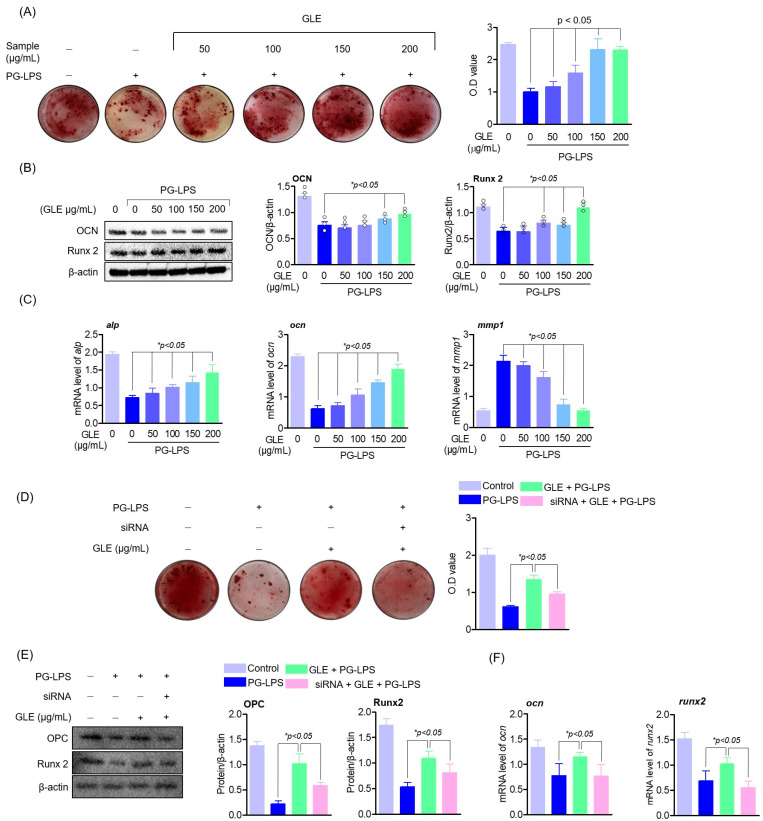
GLE regulates the osteoblastic differentiation capacity of HPDL cells lost by PG-LPS through 11β-HSD1. HPDL cells were seeded at 1 × 10^4^ cells/well in 24-well or 6-well plates and treated with PG-LPS together with GLE treatment in osteoblast differentiation media (containing 50 µg/mL ascorbic acid, 0.1 µM dexamethasone, and 10 mM β-glycerophosphate) to induce osteogenesis for 14 days. (**A**) Alizarin Red S staining (24-well full hole) was performed to confirm whether the indicated concentration of GLE induced mineralization in HPDL cells. (**B**) Western blot analysis was performed to confirm the expression of osteoblast-specific (OCN, Runx2) proteins. (**C**) Real-time PCR analysis was performed to confirm the levels of osteoblast-specific genes (*alp and ocn*) and matrix-degrading enzyme (mmp1). (**D**) Alizarin Red S staining was performed with or without silencing 11β-HSD1 with siRNA. (**E**,**F**) The levels of osteoblast-specific proteins or genes were analyzed with or without silencing 11β-HSD1 with siRNA. Data are expressed as mean ± SD. * *p* < 0.05, compared with the group treated with PG-LPS only or GLE + PG-LPS treated group.

Inhibiting the periodontal inflammatory response and restoring the function of periodontal tissue is known as another important treatment strategy for periodontitis. Therefore, to evaluate the effect of GLE on the function of periodontal tissue, the effect of HPDL cells on inducing osteoblast differentiation was evaluated. First, HPDL cells were treated with GLE at the indicated concentrations simultaneously with the induction of osteoblast differentiation and treated with PG-LPS for 7 days. As a result, GLE effectively restored the osteoblastic differentiation ability of HPDL cells inhibited by PG-LPS through the formation of mineralized nodules (Figure 6A); the gene expression of alkaline phosphatase (*alp*) and osteocalcin (*ocn*), which are osteoblast differentiation-specific markers that play an important role in early osteoblast formation were effectively protected and restored (Figure 6B,C). Therefore, we sought to evaluate the effect of 11β-HSD1 on the osteoblast differentiation recovery effect of GLE, not only in the anti-inflammatory process of GLE shown above but also in the process of inducing lost osteoblast differentiation. Research results show that, as with the anti-inflammatory effect of GLE, the silencing of 11β-HSD1 reverses the osteoblast differentiation-inducing effect of GLE shown previously and also reverses osteoblast differentiation-specific protein expression and gene levels (Figure 6D,E). These results suggest that GLE in HPDL cells stimulated with PG-LPS not only controls periodontal inflammation but also contributes to the treatment of periodontitis by regulating the osteoblastic differentiation of periodontal ligament cells, which plays a pivotal role in periodontal health. It is believed that this 11β-HSD1 regulatory effect of GLE also plays an important role in the osteoblast differentiation process.

### 2.7. GLE Suppresses Oxidative Stress Induced by PG-LPS Through Regulating 11β-HSD1

**Figure 7:**
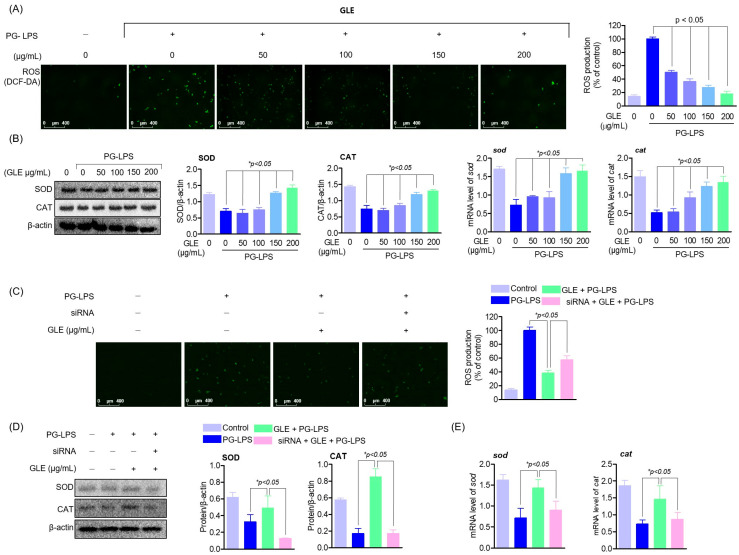
GLE suppresses oxidative stress induced by PG-LPS through regulating 11β-HSD1. (**A**) DCF-DA staining was performed to determine whether the indicated concentrations of GLE inhibit ROS production by PG-LPS treatment. (**B**) Superoxide dismutase (SOD) and catalase (CAT) levels were measured by western blot analysis and real-time PCR analysis. (**C**) GLE regulates ROS with or without silencing 11β-HSD1 by siRNA. The effects of 11β-HSD1 on the SOD and CAT regulatory effects of GLE were evaluated. (**D**,**E**) The effects of 11β-HSD1 on the SOD and CAT regulatory effects of GLE were confirmed through western blot analysis and real-time PCR analysis using the same test method. Data are expressed as mean ± SD. * *p* < 0.05, compared with the group treated with PG-LPS only or GLE + PG-LPS treated group.

Excessive generation of ROS induces protein oxidation to damage tissues, and the generation of ROS in periodontal tissues has also been known to expand the lesions of periodontitis. Therefore, the effects of GLE on ROS generated in HPDL cells stimulated with PG-LPS were evaluated. In the study results, GLE down-regulated the level of ROS generated by PG-LPS (Figure 7A) and inhibited the loss of expression levels of antioxidant proteins such as superoxide dismutase (SOD) and catalase (CAT) (Figure 7B). Additionally, 11β-HSD1 silencing again reversed the down-regulating effect of GLE on ROS generation (Figure 7C) and also reversed the down-regulated expression and gene levels of antioxidant proteins (Figure 7D,E). These results suggest that GLE regulates the ROS induced by PG-LPS, exerts an antioxidant effect, and plays an important role in the inflammation suppression strategy in the process of periodontitis lesions through oxidative stress. In addition, this suggests that the antioxidant process of GLE is also regulated through 11β-HSD1.

### 2.8. Protective Effect of GLE on Alveolar Bone and Periodontal Inflammation Lost with PG-LPS in a Periodontitis-Induced In Vivo Model

**Figure 8:**
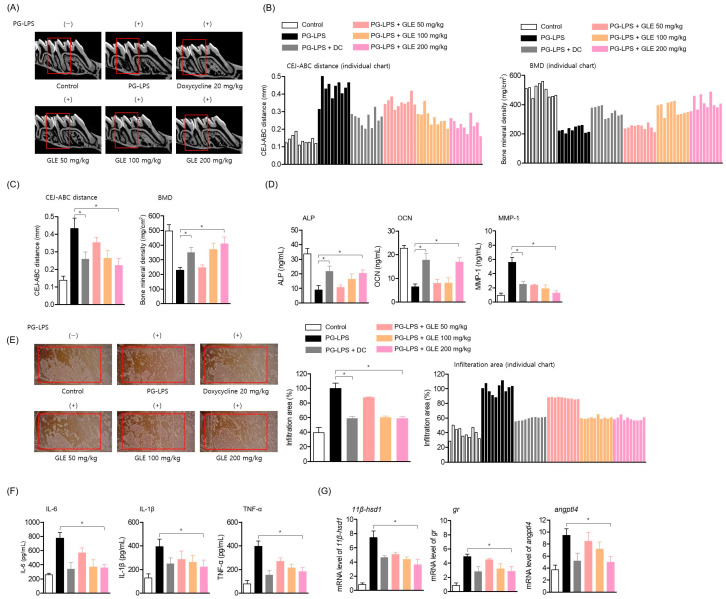
Protective effect of GLE on alveolar bone and periodontal inflammation lost with PG-LPS in a periodontitis-induced in vivo model. (**A**–**C**) PG-LPS was injected between the first and second molars for 5 days, and the indicated concentrations of GLE (50, 100, and 200 mg/kg) or positive control doxycycline (20 mg/kg) were administered orally. Additional oral administration was performed for 14 days after PG-LPS injection. After that, micro-CT images were taken and analyzed using CTAn software (Bruker 3D.SUITE, BMD, CEJ-ABC distance). (**D**) The osteoblastic differentiation-specific markers (ALP and OCN) and matrix metalloproteinase (MMP-1) lost by PG-LPS were measured using an ELISA kit from rat serum. (**E**) In addition, periodontal histological analysis was performed using hematoxylin and eosin (H&E) staining, and the inflammatory infiltration in the analysis area was compared with the PG-LPS alone treatment group and expressed as a percentage. (**F**) The pro-inflammatory cytokines (IL-6, IL-1β, and TNF-α) increased by PG-LPS were evaluated using an ELISA kit from rat serum. (**G**) The effect of GLE on the gene levels of 11β-hsd1, gr, and angptl4 increased by PG-LPS treatment was evaluated by real-time PCR analysis. Data are expressed as mean ± SD. * *p* < 0.05, compared with the group treated with PG-LPS. Red box: Region of interest.

One of the main treatment strategies for periodontitis is not only the suppression of periodontitis-causing bacteria but also the recovery or protection of damaged periodontal tissue. Therefore, the recovery effect of GLE on PG-LPS was previously shown during the osteoblast differentiation process of HPDL cells, and the periodontal tissue protection and recovery effect of GLE was researched against PG-LPS, one of the main causative bacteria of periodontitis in vivo. As a result of the study, it was confirmed through micro-CT that bone was lost in the tooth root area between the first and second molars due to PG-LPS injection, and the alveolar bone in the tooth root area lost by oral administration of GLE was recovered (Figure 8A). In addition, in the CEJ-ABC analysis, which is a major indicator of periodontal tissue recession caused by periodontitis, GLE suppressed the recession of loose periodontal tissue, and in the actual BMD analysis, it showed the effect of restoring or protecting the alveolar bone (Figure 8B,C). In addition, GLE restored ALP and OCN, specific markers of periodontal tissue production and osteoblast differentiation lost by PG-LPS, and conversely inhibited the production of MMP-1, known as the main tissue-destroying protease in periodontal disease (Figure 8D). In order to directly evaluate the effect of GLE on periodontitis, the inflammatory infiltration area of the tissue was analyzed through H&E staining, and the results showed that GLE suppressed inflammatory infiltration in a concentration-dependent manner, and in fact, the main cause of periodontitis increased by PG-LPS administration (Figure 8E). It was confirmed that the production of inflammatory cytokines IL-6, IL-1β, and TNF-α was suppressed (Figure 8F). Also, oral administration of GLE down-regulated the increased production of 11β-hsd1, gr, and angptl4 in a concentration-dependent manner, and the previously shown in vitro study results supported the claim of the research results regarding the 11β-HSD1 regulatory effect of GLE (Figure 8G). In addition, oral administration of GLE showed a general increase in body weight of experimental animals during the 20-day experimental period, and no toxicity or mortality was observed (Figure S1). As a result, the overall in vivo study results showed that GLE had a relatively superior therapeutic effect compared to doxycycline, which was used as a positive control.

### 2.9. Effect of GLE on Alveolar Bone and Periodontal Inflammation in Ligature-Induced Periodontitis-Induced In Vivo Model

**Figure 9:**
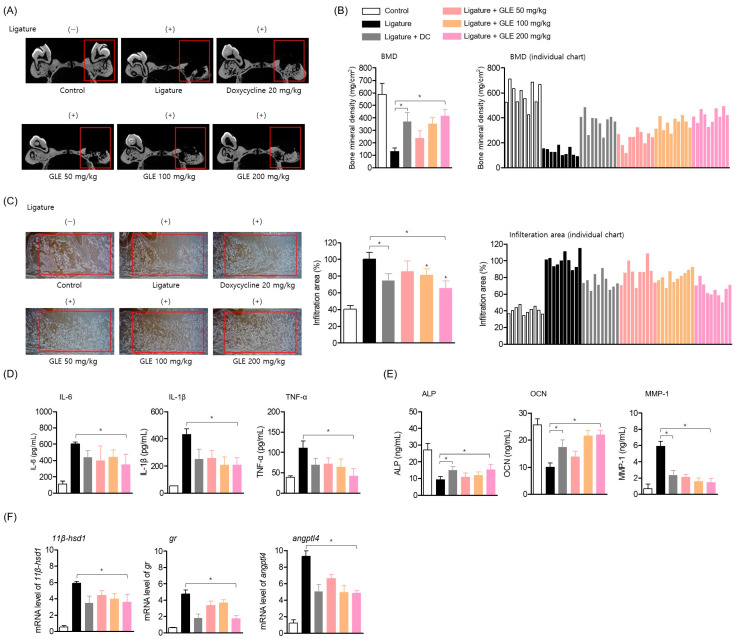
Effect of GLE on alveolar bone and periodontal inflammation in ligature-induced periodontitis-induced in vivo model. (**A**,**B**) First molar ligature was induced for 5 days, and GLE (50, 100, and 200 mg/kg) or doxycycline (20 mg/kg) as a positive control was administered orally at indicated concentrations. Teeth were extracted along with ligature removal, and oral administration was administered for an additional 8 days. After that, micro-CT images were taken and analyzed using CTAn software (BMD). (**C**) In addition, periodontal histological analysis was performed using hematoxylin and eosin (H&E) staining, and the inflammatory infiltration of the analyzed area was expressed as a percentage compared to the ligature-only group. (**D**) Increase by ligature pro-inflammatory cytokines (IL-6, IL-1β, and TNF-α) were assessed using ELISA kits in mouse serum. (**E**) Osteoblast differentiation-specific markers (ALP and OCN) and stromal metalloproteinase (MMP-1) were measured in rat serum using an ELISA kit. (**F**) The effect of GLE on the increased 11β-hsd1, gr, and angptl4 gene levels by PG-LPS treatment was analyzed by real-time PCR. Data are expressed as mean ± SD. * *p* < 0.05, compared with the group treated with ligature.

The effect of GLE was also evaluated in the ligature guidance method, which is the most used model along with the PG-LPS guidance method in general periodontitis research to date. In the study results, it was confirmed through micro-CT analysis that GLE effectively suppressed the size of the periodontal pocket formed in the tooth area extracted after ligature (Figure 9A), and GLE also induced the formation or recovery of lost alveolar bone in BMD analysis (Figure 9B). In addition, H&E analysis showed that the inflammatory infiltrated area of periodontal tissue formed by ligature was recovered (Figure 9C) and the level of pro-inflammatory cytokines increased by ligature was down-regulated (Figure 9D). In addition, GLE showed the effect of recovering osteoblast differentiation induction markers lost by ligature (Figure 9E) and suppressing the increased expression of 11β-HSD1 (Figure 9F). In the ligature-induced model, no toxicity or mortality of experimental animals were observed when GLE was administered orally (Figure S2). These research results suggest that GLE has a therapeutic effect not only on the inflammatory situation induced by PG-LPS but also on the periodontal inflammation induced by the plaque formed by ligature, and is also evidence to support the results of the in vitro study.

## 3. Discussion

Although various surgical and drug therapies have been implemented to control periodontal disease, they have not been a fundamental treatment strategy for periodontal disease. In addition, many drugs such as antibiotics and non-steroidal anti-inflammatory drugs are currently being used to treat periodontal disease, but side effects occur with long-term use, and they are not new treatment strategies [27]. Therefore, for fundamental treatment, research is being actively conducted to discover new targets that can control periodontal disease and to explore natural materials that are relatively safe from side effects [28]. Previously, it was known that chronic periodontitis triggers a hypothalamic–pituitary–adrenal (HPA) axis response, resulting in increased levels of corticosterone (the major glucocorticoid in rodents), especially glucocorticoid hormones such as 11β-HSD1 [29,30]. However, more research has been conducted on metabolic diseases including visceral fat obesity, dyslipidemia, insulin resistance, and hypertension rather than periodontitis. It has been reported that glucocorticoids play a role in experimental periodontitis, but their role in the periodontal inflammation situation and periodontal tissue has not yet been elucidated [31]. Therefore, in this study, we aimed to evaluate the effect of 11β-HSD1 regulation of GLE in periodontal inflammation.

Periodontitis is a disease caused by inflammation in the periodontium, a tissue that mainly supports teeth. In the process of periodontitis, chemokines such as CXCL-8, CXCL-1, and CCL-5 and cytokines such as TNF-α, IL-1β, and IL-6 are present, due to the action of these multi-factors, it progresses to severe periodontitis with loss of periodontal support structures [32,33]. In the inflammatory process of periodontitis, the activation of 11β-HSD1 enhances cytokines, oxidative stress, apoptosis, and mitochondrial damage through the NF-κB and MAPK signaling pathways, and further aggravates the lesions of periodontitis [34]. In particular, treatment with U0126, an ERK inhibitor, has been reported to directly down-regulate 11β-HSD1 protein expression in airway smooth muscle cells [35]; the results of this study suggest that GLE induces a decrease in 11β-HSD1 by inhibiting the phosphorylation of MAPK proteins. Also, it has been known that the regulation of these inflammatory cytokines plays an important role in periodontal inflammation as well as alveolar bone formation. Among them, cytokines such as TNF-α, IL-1β, and IL-6 are highly expressed in patients with periodontitis and promote bone resorption by directly or indirectly supporting not only inflammation but also soft tissue degradation, lymphocyte promotion, and osteoclast formation [36]. In particular, it was found that IL-6 is present in the apical end and produced by a subpopulation of neutrophils residing in the apical lesion and can directly induce osteoclast formation and bone resorption in the periodontal tissue [37,38]. Thus, this study showed that GLE effectively down-regulated the expression of pro-inflammatory cytokines and mediators, including IL-6, in experimental periodontitis-inducing models both in vitro and in vivo, and that this anti-inflammatory effect of GLE restored the lost osteoblastic differentiation capacity of HPDL cells. These results suggest the possibility that GLE may not only regulate 11β-HSD1 but also the activity of upstream signal regulators MAPK, and ultimately appears to have an effect of protecting periodontal tissue by regulating periodontal inflammation.

Generally, the production of ROS stimulates cell proliferation and differentiation in various cell lines, but excessive ROS production overwhelms the antioxidant defense system, causing cell damage and periodontal destruction due to oxidative stress [39]. The production of these ROS is directly or indirectly related to tooth soft tissue destruction and bone resorption and is known to be related to increased expression of pro-inflammatory cytokines. Therefore, studies on the protective effects of periodontitis and alveolar bone destruction through the regulation of nuclear factor erythroid-2-related factor 2 (Nrf2) have recently been reported [40,41]. In addition, recent studies have shown that the inhibition of oxidative stress due to the knockout of 11β-HSD1 can have a therapeutic effect on myocardial dysfunction caused by sepsis; 11β-HSD1 is recognized as a regulator of various diseases as a target for controlling not only inflammation but also oxidative stress [42]. The results of this study showed that the regulatory effect of GLE’s ROS regulation and antioxidant enzyme proteins (SOD, CAT) was reversed by silencing 11β-HSD1, this suggests that GLE exerts an antioxidant effect through regulating 11β-HSD1 from oxidative stress generated by periodontitis. Additionally, these results demonstrate an unknown relationship between 11β-HSD1 and oxidative stress in the periodontitis process.

Thus, in this study, GLE suppressed the expression level of pro-inflammatory cy-tokines in PG-LPS-induced human periodontal ligament cells and periodontitis-induced experimental animal models, and induced osteoblast differentiation in human periodontal ligament cells as well as specific osteoblast differentiation. The expression levels of markers ALP and OPN were increased. In addition, it restored the amount of alveolar bone along with the lost periodontal tissue in two experimental animal models inducing periodontitis and showed a therapeutic effect on inflammatory infiltration. These results suggest that GLE may play an important role in the recovery of tissue damage caused by early periodontitis by restoring alveolar bone loss, which can be found in chronic severe periodontitis, and that it will act together with the result of suppressing inflammatory infiltration due to the accumulation of inflammatory cells. Also, in the context of periodontal inflammatory lesions, GLE has been shown to act as an effective regulator of ROS production. These results suggest that GLE protects lost periodontal tissue by suppressing periodontal inflammation, suggesting a novel biological activity of GLE that has not been previously reported.

Although in this study, the in vitro study results suggest that the periodontitis regulating effect of GLE is due to 11β-HSD1, its direct effect in in vivo animal models should be further elucidated. In addition, 11β-HSD1 has been known as a major pathological target of inflammatory responses and metabolic diseases such as diabetes and obesity, and, in particular, diabetes and periodontitis are known to show a close interaction [12]. Therefore, the relationship of 11β-HSD1 between these diseases or the role of 11β-HSD1 in diabetic periodontitis still needs to be further studied.

## 4. Materials and Methods

### 4.1. Sample Preparation and Extraction

Dried *G. littoralis* F. Schmidit ex Miq leaves were harvested from Yeongdeok-gun, Gyeongsangbuk-do, Republic of Korea and extracted twice with 20-fold volume of 70% ethanol at 85 ± 5 °C for 6 h. The extract was passed through a 50-mesh filter and evaporated to obtain an extract containing about 20% solid contents (or 20% brix) in vacuo at 60 °C. The product was spray-dried with dextrin and yielded 30 ± 5%. The dried powder was used in experiments.

### 4.2. Component Analysis of GLE

GLE powder was dissolved at a concentration of 10 mg/mL in 70% Ethanol and filtrated through a 0.2 um polyvinylidene fluoride (PVDF) membrane filter. Standard of chlorogenic acid (C3878; Sigma-Aldrich, St. Louis, MO, USA) and rutin (PHL89270; Phytolab, Vestenbergsgreuth, Middle Franconia, Germany) was dissolved in 70% ethanol and used for analysis. The GLE was analyzed using high-performance liquid chromatography (Agilent Technologies 1260 Infinity II system, Santa Clara, CA, USA), and a Inertsil^®^ ODS-3 column (4.6 × 250 mm, 5 µm, GL Sciences, Torrance, CA, USA) was used. The temperature was maintained at 30 °C, with an injection volume of 10 uL at a flow rate of 1.0 mL/min. The mobile phase was composed of acetonitrile (A) and water containing 0.1% trifluoroacetic acid (B). The gradient elution conditions were as follows: initial 0–10 min A:B (14:86, *v*/*v*), 12 min A:B (20:80), 22 min A:B (30:70), 25 min A:B (100:0), 30 min A:B (100:0), and 35 min A:B (14:86). The detection wavelength was 326 nm.

### 4.3. Reagents and Antibodies

For cell culture, Minimum Essential Medium-Alpha (α-MEM), fetal bovine serum (FBS), and trypsin-ethylene diamine tetra acetic acid (EDTA) culture reagents were purchased from Wellgene (Gyeongsan-si, Republic of Korea). In addition, 3-(4,5-Dimethylthiazol-2-yl)-2,5-diphenyltetrazoliumbromide (MTT) for cell viability analysis was purchased from Amresco Inc (Cleveland, OH, USA), and for anti-inflammatory effect evaluation, TNF-α, IL-6, and IL-1β ELISA (enzyme-linked immunosorbent assay) kits were purchased from R&D system (Minneapolis, MN, USA) and analyzed according to the manufacturer’s instructions. PG-LPS used as a stimulus was purchased from InvivoGen (San Diego, CA, USA). For protein analysis, mouse monoclonal antibodies iNOS, COX-2, 11β-HSD1, GR, ANGPTL4, OCN, RUNX2, SOD, and CAT secondary antibodies were purchased from Santa Cruz Biotechnology Inc. (Dallas, TX, USA). Hybond ECL Nitrocellular membrane (Amersham Pharmacia Biotech Inc., Piscataway, NJ, USA) was purchased. Alizarin Red S, ascorbic acid, dexamethasone, and β-glycerophosphate were purchased from Sigma-Aldrich (St. Louis, MO, USA). 11β-HSD1 siRNA plasmid was purchased from Santa Cruz Biotechnology Inc. (Dallas, TX, USA). Opti-MEM and Lipofectamine RNAiMAX reagents were purchased from Invitrogen (Carlsbad, CA, USA).

### 4.4. Cell Culture

Human periodontal ligament cells (HPDL) were provided and used by Kyungpook National University (Daegu, Republic of Korea) and were reviewed and approved by the Kyungpook National University Institutional Review Board (KNU 2017-78). Isolation of cells was obtained from donor third molars as previously reported [16]. Briefly, HPDL cell culture was performed as follows: PDL was carefully separated from the root surface, and then cultured with 3 mg/mL type 1 collagenase (Worthington Biochem, Freehold, NJ, USA) and 4 mg/mL dispase (Roche, Mannheim, Germany) solution for 1 h. PDL samples from different individuals were pooled, and single-cell suspensions were obtained by passing the cells through a 70 μm strainer (Falcon, BD Labware, Franklin Lakes, NJ, USA), and then the single-cell suspensions (1 × 10^4^ cells) were seeded onto 10 cm culture plates. After that, the culture medium was cultured in α-MEM supplemented with 10% (*v/v*) fetal bovine serum (FBS) and 1% penicillin/streptomycin (Gibco BRL, Grand Island, NY, USA), and the cells were maintained at 37 °C in a humidified atmosphere of 5% CO_2_.

### 4.5. Cell Viability and Confluency

To investigate cell viability, HPDL cells were seeded in a 96-well plate at a density of 5 × 10^2^ cells/well, and cultured for 24 h, followed by treatment with indicated concentrations of GLE in 7 days. After that, cell counting was performed using the Incucyte^®^ Live-Cell Analysis System (Göttingen, Germany). For cell counting, cell counts in the stained control group were converted to percentages and averaged for the GLE treatment group. In addition, cells were cultured in the same way and treated with GLE, and 5 mg/mL MTT (3-[4,5-dimethylthiazol-2-yl]-2,5-diphenyl tetrazolium bromide) was added to 50 μL of cell suspension and cultured for 4 h. The medium was removed and 100 μL dimethyl sulfoxide (DMSO) was added. The absorbance of the dissolved formazan crystals was measured at 540 nm with a microplate reader (Tecan Trading AG) (Männedorf, Switzerland). Similarly, trypan blue (Sigma-Aldrich, St. Louis, MO, USA) staining was performed to count the number of positive cells, the number of cells in the control group was converted to a percentage, and the number in the GLE treatment group was averaged.

### 4.6. ROS Production

Reactive oxygen species (ROS) was measured using H2DCFDA (Sigma-Aldrich, St. Louis, MO, USA) and after treatment with GLE at the indicated concentrations. To determine intracellular ROS levels, HPDL cells were cultured in 6-well plates for 24 h, then treated with all concentrations of GLE (50, 100, 150, and 200 μg/mL), and then treated with 10 μg/mL PG-LPS and incubated for 2 h under humidified conditions (37 °C, 5% CO_2_). After incubation, medium containing 10 μM DCF-DA was treated to each well and incubated under humidified conditions (37 °C, 5% CO_2_). Cells were then rinsed twice with PBS and measured using the Incucyte^®^ Live-Cell Analysis System (Sartorius, Göttingen, Germany).

### 4.7. Alizarin Red S Staining (Mineralization Assay)

After seeding HPDL cells at 1 × 10^4^ cells/well in a 6-well plate, osteogenesis was induced for 14 days in an osteoblast differentiation medium (containing 50 µg/mL ascorbic acid, 0.1 µM dexamethasone, and 10 mM β-glycerophosphate). Afterwards, the cells were fixed with 4% polyformaldehyde for 30 min and stained with 0.1% Alizarin Red S at room temperature for 1 h to stain the calcified nodules, and then observed under an optical microscope (Nikon, Tokyo, Japan) to photograph the calcified nodules. To quantify the amount of calcium accumulation in the calcified nodules, the calcium was dissolved in 10% cetylpyridinium chloride (CPC) and the absorbance was measured at 560 nm using a multifunctional microplate reader (M1000 Pro, TECAN, Männedorf, Switzerland), referring to the method of a previously reported study [43].

### 4.8. Fluorescein Isothiocyanate (FITC) Staining

To confirm knockout of 11-βHSD1 in HPDL cells, fluorescein isothiocyanate (FITC) analysis was performed after transfection. After transfection, cells were fixed by treating with 4% formaldehyde solution for 30 min and then cultured overnight at 4 °C using 11β-HSD1 antibody (Santa Cruz Biotechnology Inc., cat. no. sc-518168, Dallas, TX, USA). The following staining was performed. Afterwards, cells were photographed using the Incucyte^®^ Live-Cell Analysis System.

### 4.9. Detection of mRNA Levels by Real-Time Quantitative PCR

Total RNA was extracted using TRIzol/chloroform reagent (Bioneer, Daejeon, Republic of Korea) according to the manufacturer’s instructions, and reverse transcription of RNA was performed using the RT PreMix kit (Enzynomics, Daejeon, Republic of Korea). cDNA was then amplified with SYBR Premix Ex Taq (Takara, Tokyo, Japan) and 40 cycles of 50 °C for 2 min, 95 °C for initial denaturation for 10 min, 95 °C for 15 s, and 60 °C for annealing for 30 s were performed. Each target mRNA analyzed was normalized to the cycle threshold (Ct) value of GAPDH as a housekeeping gene. Primers for each gene used in this study were provided by Bioneer Co., Ltd. (Daejeon, Republic of Korea), and the sequences of the primers are shown in Table 2.

### 4.10. Western Blot Analysis

The expression levels of each target protein were analyzed using Western blot analysis, and the experimental method is as follows. HPDL cells were washed with PBS and lysed in RIPA lysis buffer (50 mM Tris pH 8.0, 150 mM NaCl, 0.02% sodium azide, 0.2% SDS, 1 mM PMFS, 10 μL/mL aprotinin, 1% igapel 630 (Sigma-Aldrich, St. Louis, MO, USA)), and protein samples were extracted and centrifuged at 23,000× g for 15 min. The protein supernatant was quantified using a protein assay kit (Bradford Reagent, Sigma-Aldrich, St. Louis, MO, USA). Equal amounts of protein (20 μg) were then separated by 12% SDS-PAGE and transferred to PVDF membranes. The membranes were washed with 5% (*w*/*v*) skim milk for 1 h at room temperature. After blocking, the membranes were rinsed with TBST buffer for 1 h and then incubated with primary antibodies overnight at 4 °C. The membranes incubated with primary antibodies were washed with Tris-buffered saline (TBS-T) buffer containing Tween 20 and then incubated with secondary antibodies. Protein bands were detected using Enhanced Chemiluminescence (ECL) Western blotting detection reagents (Thermo Fisher Scientific, Waltham, MA, USA). Band images were scanned with a ChemiDoc XRS+ system (Bio-Rad, Hercules, CA, USA). The detected bands were analyzed and quantified using Image J version 1.54i software (National Institutes of Health, Bethesda, MD, USA).

### 4.11. Serum Cytokines ELISA

Each inflammatory cytokine expression was analyzed according to the instructions of the kit manufacturer, R&D Systems (Minneapolis, MN, USA). After the experiment was completed, blood samples were collected from the veins of the mice. The samples were then centrifuged and centrifuged at a low temperature of 4 °C. Serum was separated. The separated blood crystals were sealed and stored at −20 °C until analysis. Serum collected from mice in each group was used in an enzyme-linked immunosorbent assay (ELISA) kit (according to the manufacturer’s instructions). The analysis was performed using a 96-well plate (R&D system, NE, Minneapolis, MN, USA) as follows: For each cytokine analysis, each antibody was incubated in a 96-well plate, and then each well was washed with washing buffer. After that, the diluent was added and incubated at room temperature for 1 h, then the sample was treated and incubated at room temperature for 3 h. After that, a second wash was performed and treated with Streptavi-din-HRP B solution and incubated for 30 min. Afterwards, each substrate solution was placed in a well plate, incubated at room temperature, protected from light, and analyzed at 540 nm wavelength using a plate reader (Tecan Trading AG) (Männedorf, Switzerland).

### 4.12. Transfection with siRNA

HPDL cells were transfected with Opti-MEM and Lipofectamine RNAiMAX reagent (Invitrogen, Carlsbad, CA, USA) were mixed and transfected with 50 nM 11β-HSD1 siRNA (Santa Cruz Biotechnology Inc., cat. no. sc-41377, Dallas, TX, USA), and the cells were transfected for 48 h, after that, they were used in the experiment.

### 4.13. Animals

All animal experimental procedures were approved by the Animal Care Committee of Chungnam National University (202308-CNU-334) and were performed in accordance with the “Guidelines for Animal Use” established by the Animal Care Committee of Chungnam National University (Daejeon, Republic of Korea). Briefly, the experimental animals and their housing environment were described. Eight-week-old male Spra-gue-Dawley rats were purchased from Samtako Inc. (Osan, Republic of Korea) and were maintained in the Community Animal Laboratory of Chungnam National University (Daejeon, Republic of Korea). They were isolated and acclimated in stainless steel wire cages (260 W × 350 D × 210 H mm) for 1 week before the experiment, and the housing environment was maintained at a temperature of 21.0–23.1 °C and a relative humidity of 30–50%. In addition, the lighting time was 12 h/day (7:00 a.m.–7:00 p.m.), and the illuminance was 150–300 lux. The feed used was solid feed for laboratory animals (Harlan Laborato-ries, Inc., Greenfield, IN, USA), and the solid feed was placed in a feeding trough so that the animals could consume it freely. The drinking water was filtered using a filter sterilizer and then irradiated with ultraviolet rays from Cheongju City tap water and supplied freely using an automatic watering device.

### 4.14. Ligature-Induced Periodontitis Inflammation Model

**Scheme 1:**
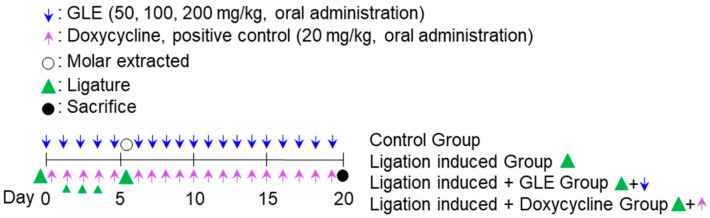
Ligature-induced experimental each group and schedule.

In the experimental model of ligature-induced periodontitis, GLE was administered orally. Eight-week-old male Sprague-Dawley rats (each group *n* = 10, total of 60 rats, each group: control, ligation-induced group, ligation + doxycycline 20 mg/kg, ligation + oral administration of GLE 50 mg/kg, ligation + oral administration of GLE 100 mg/kg, ligation + oral administration of GLE 200 mg/kg) were anesthetized by CO_2_ in-halation. Except for the control group, the maxillary right first molar was fixed and ligated with a non-absorbable silk knot for 5 days. The ligation site was checked daily to determine whether periodontitis was induced, and ad libitum food was administered. During the ligation period of 5 days, GLE and doxycycline, a positive control, were administered orally at the indicated concentrations to each group. On the 5th day after ligation, the silk knot was removed, the first molar was extracted, and hemostasis was performed on the periodontal pocket formation site. After that, GLE and doxycycline were administered orally to each group for 14 days, and the oral administration was continued for a total of 19 days including the ligation period. All experimental animals were sacrificed on the 20th day after the end of the experiment, and the maxillary cranial bones were extracted (Scheme 1).

### 4.15. PG-LPS Induced Periodontal Inflammation

**Scheme 2:**
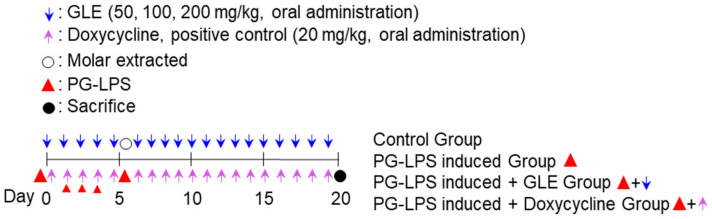
PG-LPS induced experimental each group and schedule.

For periodontitis induction using PG-LPS, 8-week-old SD rats were divided into each group (each group *n* = 10, total 60, each group: control group, PG-LPS-only induced group, PG-LPS + doxycycline 20 mg/kg, PG-LPS + GLE 50 mg/kg, PG-LPS + GLE 100 mg/kg, PG-LPS + GLE 200 mg/kg). After that, each group except the control group was anesthetized by CO_2_ inhalation and PG-LPS was injected daily between the first and second molars for 5 days, and at the same time, GLE at the indicated dose and doxycycline as a positive control were orally administered for 5 days. After the completion of PG-LPS induction for 5 days, GLE at the indicated dose and doxycycline as a positive control were orally administered to each group for 14 days. Similarly, GLE and doxycycline were administered for a total of 19 days, and after the end of the experiment, the experimental animals were sacrificed, and the maxillary skull was extracted. Similarly, GLE and doxycycline were administered for a total of 19 days, and at the end of the experiment on the 20th day, the experimental animals were sacrificed, and the maxillary skulls were removed (Scheme 2).

### 4.16. Micro-CT Imaging and Analysis

Micro-CT analysis was performed on sacrificed experimental animals, and the experimental methods and conditions are as follows. Bone mineral density analysis (pixel size 10 μM) was performed using tube current (160 μA), tube voltage (90 kVp), imaging time (180 s), pixel size (10 μM), and FOV (rectangular volume of alveolar bone (400 μm width × 500 μm thickness × 1400 μm height) next to the mesial and distal roots) from the accumulated maxillary skull. In addition, the raw data obtained from the coronal section by changing direction were loaded into CTAn, and the images were scanned to calculate the BMD based on the above threshold. In addition, the alveolar bone excluding the root of the cemento-enamel junction was set as the region of interest (ROI) using the alveolar bone interpolation method, and VGStudio MAX 1.2.1 software (Volume Graphics, GmbH, Heidelberg, Germany) was used for ROI analysis. Data were analyzed by mean ± SD and one-way analysis of variance in SPSS Statistics 19.0 software (Armonk, NY, USA).

### 4.17. Histological Staining

To perform hematoxylin-eosin (H&E) staining on periodontal tissues obtained from the extracted maxillary skull, the extracted periodontal tissues were fixed in 10% formalin, embedded in paraffin, cut into 5 μm sections, and mounted on slides. H&E staining was then performed on the fixed slides. Tissue infiltration was observed from the stained slides using a fluorescence Olympus IX microscope 71-F3 2PH (Tokyo, Japan), and the same region of interest was set at the periodontal extraction site of all samples and compared with the ligation-induced group. The area of white soft tissue was expressed as a percentage compared to the control group.

### 4.18. Measurements of CEJ-ABC

The area from the CEJ (cement-enamel junction) between the fixed maxillary second and third molars to the alveolar bone ridge (ABC) was set as the region of interest for analysis, and the linear areas measured were the buccal palate, palatine palate, mesio-buccal, and the mesial-palatal areas were selected as measurement areas. Then, the distance from the furthest root of the second and third molars to the top of the teeth was measured (in millimeters, measured only in the PG-LPS-induced model).

### 4.19. Statistics

Each experiment was independently repeated three times for reproducibility, and the results were expressed as the mean ± standard deviation (S.D.). Statistical analysis was performed using SPSS Statistics 19.0 software (Armonk, NY, USA), and differences between groups were analyzed using one-way analysis of variance (ANOVA) followed by Tukey’s test or Student’s *t*-test. Statistical significance was determined as *p*-value < 0.05.

## 5. Conclusions

In this study, the effect of GLE on periodontitis was evaluated using in vitro and in vivo experimental models known as representative periodontitis models. In addition, we investigated the role of 11β-HSD1, which has been known as a major target for diseases such as obesity and general inflammation, in the progression of periodontitis; in particular, through this study, we explored the new physiological activity of GLE, and in addition, the periodontitis-related activity of GLE. We demonstrated a relationship with 11β-HSD1 as a regulatory target. Therefore, we suggest the possibility of using GLE as a new periodontitis treatment agent through regulating 11β-HSD1.

## Figures and Tables

**Table 1:** Calibration curve and contents of compounds in GLE.

Analytes	Calibration Curve	r^2^	Content (mg/g)
Chlorogenic acid	y = 24.191x − 18.879	1.000	7.040 ± 0.041
Rutin	y = 17.916x − 11.975	1.000	20.194 ± 0.004

**Table 2:** Primer sequences.

Target Gene	Sequence (5′→3′)		Accession Number
*il-6*	Forward	AGTGAGGAACAAGCCAGAGC	NM_000600.4
	Reverse	GTCAGGGGTGGTTATTGCAT	
*il-1β*	Forward	AACCTCTTCGAGGCACAAGG	NM_000576.2
	Reverse	GTCCTGGAAGGAGCACTTCAT	
*tnf-α*	Forward	GCCTCTTCTCCTTCCTGATCGT	NM_000594.2
	Reverse	TGAGGGTTTGCTACAACATGGG	
*alp*	Forward	TGCAGTACGAGCTGAACAGG	NM_000478
	Reverse	GTCAATTCTGCCTCCTTCCA	
*ocn*	Forward	CGCTACCTGTATCAATGGCTGG	NM_199173
	Reverse	CTCCTGAAAGCCGATGTGGTCA	
*mmp-1*	Forward	ATGAAGCAGCCCAGATGTGGAG	NM_002421
	Reverse	TGGTCCACATCTGCTCTTGGCA	
*sod*	Forward	CTCACTCTCAGGAGACCATTGC	NM_000454
	Reverse	CCACAAGCCAAACGACTTCCAG	
*cat*	Forward	GTGCGGAGATTCAACACTGCCA	NM_001752
	Reverse	CGGCAATGTTCTCACACAGACG	
*11β-hsd1*	Forward	AAATACCTCCTCCCCGTCCT	NM_017080
	Reverse	TCCTGCCTCAACAACAAATC	
*gr*	Forward	GGAATAGGTGCCAAGGATCTGG	NM_000176
	Reverse	GCTTACATCTGGTCTCATGCTGG	
*angptl4*	Forward	GATGGCTCAGTGGACTTCAACC	NM_139314
	Reverse	TGCTATGCACCTTCTCCAGACC	
*β-actin*	Forward	AGAGCTACGAGCTGCCTGAC	NM_001101
	Reverse	AGCACTGTGTTGGCGTACAG	
*gapdh*	Forward	TGTTCGTCATGGGTGTGAAC	NM_002046
	Reverse	GTCTTCTGGGTGGCAGTGAT	

## Data Availability

The original contributions presented in this study are included in the article/Supplementary Material. Further inquiries can be directed to the corresponding author.

## Supplementary Materials

The following supporting information can be downloaded at: https://www.mdpi.com/article/10.3390/molecules30142903/s1, Figure S1. Body weight change of all experimental animals (Ligature induction method, each group *n* = 10, tatal *n* = 60). Figure S2. Body weight change of all experimental animals (PG-LPS induction method, eachgroup *n* =10, tatal *n* = 60).
