# Supplementary material for: Ameliorating Effect of Glehnia littoralis Extract on Periodontitis Through Regulation of 11β-Hydroxysteroid Dehydrogenase Type 1 in an Experimental Periodontitis Model
Source: Molecules. 2025 Jul 9;30(14):2903. doi: 10.3390/molecules30142903 (PMC12299895; doi:10.3390/molecules30142903)
Supplement: Supplementary file 1 [file molecules-30-02903-s001.zip › molecules-3623380-supplementary.pdf]

SUPPLEMENTARY MATERIAL

Ameliorating Effect of *Glehnia littoralis* Extract on Periodontitis Through Regulation of 11β-Hydroxysteroid Dehydrogenase Type 1 in an Experimental Periodontitis Model

Eun-Nam Kim [1], Nguyen Minh Trang [1], Chae Lee Park [2], Sang-Yoon Kim [2], MinKyun Na [1] and Gil-Saeng Jeong [1,*]

[1] College of Pharmacy, Chungnam National University, Daejeon 34134, Republic of Korea; enkim@cnu.ac.kr (E.-N.K.); ngminhtrang52@gmail.com (N.M.T.); mkna@cnu.ac.kr (M.N.)
[2] Healthcare Research Division, HuonsGlobal Bldg., Pangyo 13449, Republic of Korea; cherry890709@huons.com (C.L.P.); sykim78@huons.com (S.-Y.K.)
* Correspondence: gsjeong@cnu.ac.kr; Tel.: +82-42-821-5937

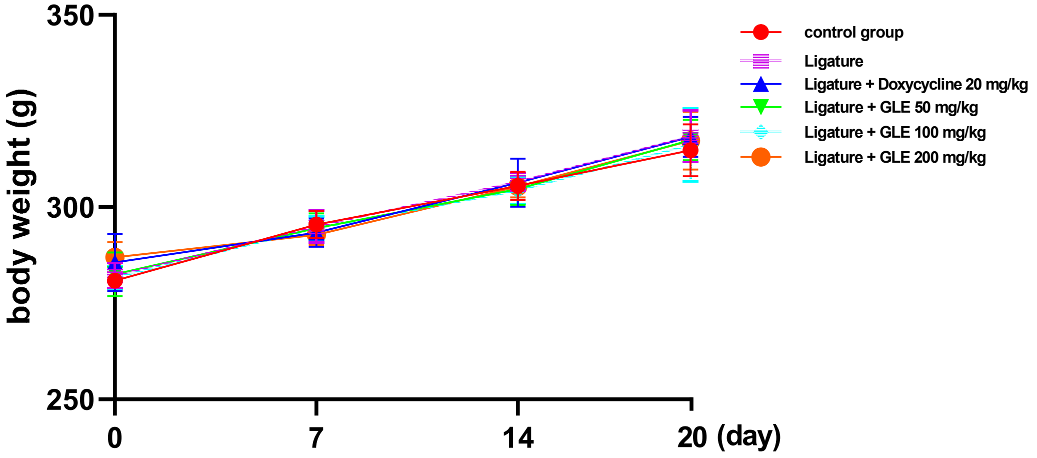

Figure S1. Body weight change of all experimental animals (Ligature induction method, each gr oup *n*=10, tatal *n*= 60).

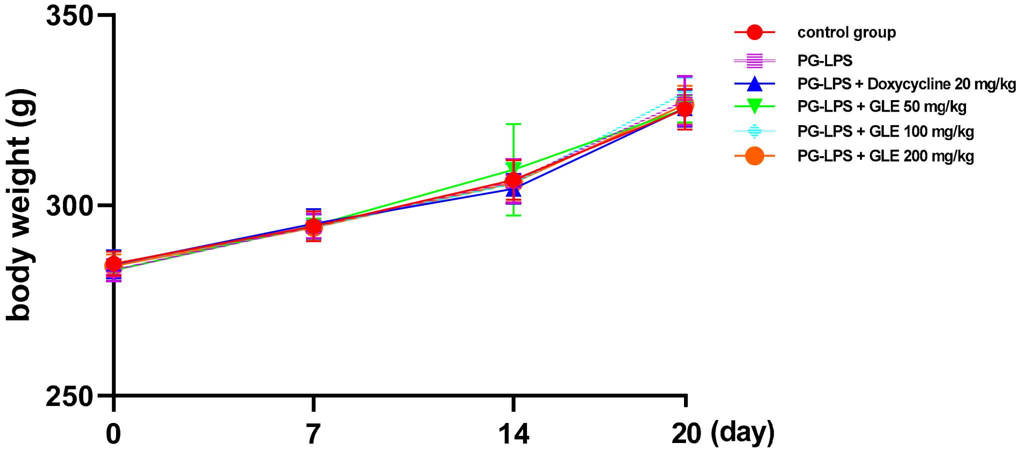

Figure S2. Body weight change of all experimental animals (PG-LPS induction method, eachgro up *n*=10, tatal *n*= 60).
